# Influence of orthodontic appliances and nitrate on the oral microbiota

**DOI:** 10.1007/s00253-025-13496-0

**Published:** 2025-05-06

**Authors:** Elisabeth Reichardt, Martin Eigenthaler, Paul-Georg Jost-Brinkmann, Angelika Stellzig-Eisenhauer, Carlalberta Verna, Iris Plumeier, Silke Kahl, Howard Junca, Ramiro Vilchez-Vargas, Dietmar H. Pieper

**Affiliations:** 1 Department of Orthodontics, University Hospital Würzburg, Würzburg, Germany; 2 Department of Orthodontics, Dentofacial Orthopedics and Pedodontics, CharitéCenter for Oral Health Sciences CC3, Charité - Universitätsmedizin Berlin, Berlin, Germany; 3 Department of Pediatric Dentistry and Orthodontics, University Center for Dental Medicine, UZB, Basel, Switzerland; 4 Microbial Interactions and Processes Research Group, Helmholtz Centre for Infection Research, Braunschweig, Germany; 5 Medical Department 2, University Hospital LMU Munich, Munich, Germany; 6 Present Address: Section of Orthodontics, Department of Odontology, Faculty of Health and Medical Sciences, University of Copenhagen, Copenhagen, Denmark

**Keywords:** Oral microbiota, Orthodontic fixed appliances, Nitrate, Gingival disease, Oral health, Dietary supplements

## Abstract

**Abstract:**

In this pilot study, we investigated the bacterial changes introduced on the subgingival, tongue, and saliva microbiota during fixed orthodontic treatment, with or without daily administration of nitrate-containing beet juice for 2 weeks in 22 individuals with good general health. We followed clinical parameters in combination with microbiota changes before, after 2 weeks, and after 6 months of treatment with fixed orthodontic appliances. In accordance with variations in community composition at the sampling sites, effects to orthodontic treatment differed. Subgingival communities responded promptly to orthodontic treatment with no additional structural changes over time, whereas saliva and tongue communities were affected only after extended treatment. Periodontal pathogens such as *Selenomonas sputigena* were enriched in subgingival communities, whereas *Streptococcus mutans* was enriched in saliva. Specifically, *Rothia mucilaginosa* increased tremendously in relative abundance in both tongue and saliva communities. The effect of beet juice on microbial composition was significant in subgingival samples even though the differences were not mirrored in single differentially distributed genera or species. This indicates changes in the complete subgingival microbial net of interacting species. However, the prevention of *Corynebacterium matruchotii* enrichment by beet juice may be important for prevention of biofilm formation. Enrichment of *Neisseria flavescens* group bacteria and *Abiotrophia* and depletion of different *Actinomyces* and *Stomatobaculum* were observed on tongue communities. We conclude that subgingival microbiota are rapidly affected by fixed orthodontic appliances and can be positively influenced by regular administration of nitrate-containing juice.

**Key points:**

*• The subgingival site, tongue, and saliva contain different microbiota*

*• The microbiota react differently to orthodontic treatment and beet juice*

*• Key genera and species affected by treatments were identified*

**Supplementary Information:**

The online version contains supplementary material available at 10.1007/s00253-025-13496-0.

## Introduction

The human oral cavity is inhabited by complex oral microbial communities, which may form biofilms on both soft and hard tissues (Kolenbrander et al. 2006). It is well established that the oral cavity comprise diverse niches, with the subgingival and supragingival plaque communities being highly different to those of other oral sites like saliva, tongue dorsum, or buccal mucosa (Eren et al. 2014) with distinct species of a genus being specialized for different oral sites (Baker et al. 2024). Fixed orthodontic therapy increases the risk of plaque accumulation around brackets and wires, thereby modifying the oral environment in terms of saliva composition, pH, and buffering capacity (Reichardt et al. 2019). The resulting clinical symptoms, such as gingival hyperplasia, swelling, and bleeding on probing, provide an ideal environment for anaerobic bacteria to build up biofilms (Kim et al. 2012; Reichardt et al. 2019). Previous studies have shown that changes in the oral bacterial community are associated with a transition from periodontal health to gingivitis and periodontal disease (Haffajee and Socransky 1994; Kumar et al. 2006). The insertion of fixed orthodontic appliances appears to be linked to a qualitative change in planktonic saliva and subgingival microbiota. Previous work used mainly plating and assays to specifically detect periodontal pathogens and could indicate an increase of *Campylobacter rectus* or *Tannerella forsythia* among others in subgingival samples (Kim et al. 2012). More recent analyses applied high-throughput sequencing to monitor total bacterial community changes and typically observed an enrichment of *Selenomonas sputigena*; however, despite the differences in community, no clear difference in responses between saliva and subgingival samples microbiota is reported yet (Chen et al. 2022; Koopman et al. 2015; Wang et al. 2024).

Products designed to prevent biofilm formation in orthodontic patients, such as interdental brushes, specialized orthodontic toothbrushes, antimicrobial mouth rinses, and toothpastes, are commercially available, but their efficacy appears to be limited during the treatment period (Rafe et al. 2006; Yaacob et al. 2014). Therefore, additional therapeutic measures might be necessary to maintain good oral health during orthodontic therapy. One promising treatment strategy includes the use of nitrate, which is naturally present in plants and abundant in various vegetables such as beets (Rosier et al. 2022). A nitrate-rich diet has been shown to affect the microbiome residing in the oral cavity (Lundberg and Govoni 2004; Rosier et al. 2021) and probably enriches for nitrate/nitrite/nitric oxide respiring bacteria. Nitrate from a nitrate-rich diet may thus be transformed by bacteria, and the swallowed nitrite may be transformed to nitric oxide which is associated with various systemic benefits, including lowered blood pressure, improved sports performance, increased mucus production, and antimicrobial effects (Lundberg et al. 2018; Rosier et al. 2022). Previous studies have demonstrated that regular nitrate administration is furthermore effective in preventing caries development, reducing dental biofilm formation, and alleviating gingival inflammation mediated by nitric oxide formation which may inhibit sensitive periodontal pathogens or mediated by limited acidification (Rosier et al. 2022). Oral microbial community changes have predominantly been analyzed in saliva and typically comprise higher levels of oral health-associated nitrate-reducing genera *Neisseria* and *Rothia* and a depletion of caries-associated genera such as *Veillonella* or *Leptotrichia* (du Toit et al. 2024; Rosier et al. 2020). Overall, nitrate intake might thus be a promising strategy for stimulating oral health (Jockel-Schneider et al. 2016; Rosier et al. 2020; Vanhatalo et al. 2018).

The aim of this study was to assess the effect of daily administration of a nitrate-containing beet juice during orthodontic therapy on the subgingival, tongue, and saliva microbiota.

## Materials and methods

### Study design

The current study was performed including 22 patients, with a mean age of 13 years undergoing orthodontic treatment with buccal fixed appliances at the Department of Orthodontics, University Hospital of Wuerzburg, Germany, or the Department of Orthodontics and Pediatric Dentistry, University Center for Dental Medicine, Basel, Switzerland (see Supplementary Table S1). The study was performed according to the Helsinki Declaration, registered at ClinicalTrials.gov (NCT06411977) and approved by the Medical Ethic Committee of the University of Wuerzburg, Germany resp. Northwest and Central Switzerland (trial register: 185–2017; 2019–01896). The randomization allocation list was made by a blinded person in Microsoft Office Excel 2003 (Microsoft, Redmond, WA, USA) using the random number generation function in the analysis tool pack for one variable with a discrete distribution, allocating 50% of the 22 subjects to the test and 50% to the control group. In all cases, participants were under 18 years of age. Therefore, the parents/legal guardians of the participants signed informed consent before participation.

### Patients and indices

Before enrollment into the study, the gingival condition of the potential participants was verified to be healthy (periodontal pockets ≤ 3.4 mm, Periodontal Screening Index (PSI) ≤ 2, where a PSI of 2 is defined as: physiological gingival pockets below 3.4 mm, the presence of moderate calculus, and moderate bleeding on probing (Chapple et al. 2018; Loe 1967)). The inclusion criteria for the study were good general health of the participants, without any medical conditions, diseases, or requirement of medication. Individuals with significant systemic disease, antimicrobial and anti-inflammatory therapy within the past 6 months, periodontal pockets > 3.4 mm, or PSI > 2, self-reported pregnancy, and smokers were excluded. Gingival conditions were assessed by Plaque Index (PI), PSI, and Papilla Bleeding Index (PBI) (Charles and Charles 1994; Loe 1967; Saxer and Muhlemann 1975).

A total of 22 subjects (10 females and 12 males) set to receive fixed orthodontic appliances in both jaws were to participate in the study (Supplementary Table S1). For the orthodontic treatment, metal brackets (Victory, 3 M Unitek, Monrovia, CA, USA) were bonded directly with composite resin (Transbond XT, 3 M Unitek, Monrovia, CA, USA) on incisors, premolars, and second molars. Molars were separated for up to 7 days before insertion of fixed orthodontic appliances. After disinfecting with ethanol and drying, the molar bands (MBT, Ormco, Orange, CF, USA) were cemented (Ketac™ Cem Easymix, 3 M Unitek, Monrovia, CA, USA) on the first molars of the upper and the lower arch. For each patient, the fixed orthodontic appliances were inserted at the same treatment appointment, and the arch wires were ligated using elastics (3 M Unitek, Monrovia, CA, USA). All participants received oral hygiene instruction as part of the standard procedure for orthodontic patients after the insertion of fixed orthodontic appliances (t1). The instruction included a demonstration of the cleaning process using a toothbrush and interdental brushes on an orthodontic model. Participants were advised to brush their teeth at least twice per day.

Eleven subjects received daily 120 mL beet juice (FITRABBIT, Voglsam, Hofkirchen, Austria) containing 72% concentrated beet juice, lactic acid fermented, 10% concentrated pomegranate juice, 10% concentrated sour cherry juice, 5% herbal spice extract (water, peppermint, lemon balm, lavender, nettle, black cumin), and 3% acerola juice from concentrate, while the control group did not receive food or drinks. This juice has a specifically high content of nitrate (3.9 g/l) compared to other commercial pure or concentrated beet juice products (Wruss et al. 2015) but only medium chloride and sulfate levels. Beet juice contains a mean of 77 g/l of sugar (mainly sucrose). It was assumed that 120 mL of juice (with roughly 8 g of sucrose) does not tremendously impact the daily sucrose intake (estimated as 80 g/day) (Linseisen et al. 1998). It was used for 14 days from the time of insertion of the fixed appliances every day after lunch. All subjects received regular oral hygiene instructions and were advised to use interproximal brushes to clean the areas of the tooth adjacent to the bracket underneath the orthodontic wire. The subjects were instructed not to clean their teeth 30 min before saliva, tongue, and subgingival samples for microbiome analysis were taken. These samples were obtained at three time-points during this study: t0 (approximately 1 week before placement of the fixed orthodontic appliances), t1 (2 weeks after placement), and t2 (6 months after placement). Saliva samples were collected using a Falcon tube (Sarstedt, Nuembrecht, Germany). Tongue samples were collected using floqswabs (Copan, Brescia, Italy), and the subgingival plaque was collected from the buccal surface of the upper right first premolars using a sterile paper point for 30 s under relative dryness (ISO 30, Dentsply, Charlotte, NC, USA) within periodontal pockets ≤ 3.4 mm. In cases where the gingival margin reached the bracket, the subgingival plaque was collected mesially and/or distally from the bracket. The samples were immediately stored at − 80 °C. Gingival indices of the subjects were recorded at visits t0, t1, and t2. Changes in gingival conditions were assessed by two-way repeated measures ANOVA and a mixed effects model in case of missing data.

### 16S rDNA amplification and sequencing

A 2-step PCR-approach was used to amplify highly discriminatory V1-V2 variable region of the 16S rRNA gene (Camarinha-Silva et al. 2014; Johnson et al. 2019). PCR with optimized primers 27 Fbif and 338R (Rath et al. 2017) containing part of the sequencing primer sites as short overhangs (given in italics) (*ACGACGCTCTTCCGATCT*AGRGTTHGATYMTGGCTCAG and *GACGTGTGCTCTTCCGATCT*TGCTGCCTCCCGTAGGAGT, respectively) was used to enrich for target sequences (20 cycles). A second amplification step of 10 cycles added the two indices and Illumina adapters to amplicons (Rath et al. 2017). Amplified products were purified, normalized, and pooled using the SequalPrep Normalization Plate (Thermo Fisher Scientific, Waltham, MA, USA) and sequenced on an Illumina MiSeq (2X300 cycles, San Diego, CA, USA). Demultiplexed raw data for all the amplicon sequencing pair-end datasets are publicly available at the NCBI Sequence Reads Archive (SRA) under BioProject accession number PRJNA1158742 (https://dataview.ncbi.nlm.nih.gov/object/PRJNA1158742?reviewer=6fu6oh2pu2i48f1obgidttpkvu).

### Bioinformatic and statistical analyses

The FASTQ files were analyzed with the dada2 package version 1.12.1 in R (Callahan et al. 2016). The quality-trimming and filtering steps were performed using the filterAndTrim function. Forward and reverse reads were trimmed on the 5′-end by 20 and 19 bases, respectively. Reads were truncated to a length of 240 bases, and a maximum of 2 expected errors per read was permitted. After denoising and paired-end reads merging, chimeras were removed. Remaining non-bacterial sequences (eukaryota, mitochondria, chloroplast) were manually deleted (Supplementary Table S2a). Sequence types were annotated based on the naïve Bayesian classification with a pseudo-bootstrap threshold of 80% using RDP set18 (Cole et al. 2014). Sequence variants were then manually analyzed against the RDP database using the Seqmatch function to define the discriminatory power of each sequence type. All annotations were then upgraded using SILVA SSU138.1 rRNA database (Quast et al. 2013). In some cases where genus or higher level annotations were distinct between SILVA SSU138 on the one hand and RDP and the LPSN database (Parte et al. 2020) on the other hand, LPSN annotations were used (e.g. differentiation of *Lactobacillus* or *Prevotella* in different genera). In addition to sequences with species level annotations available from RDP, 16S rDNA sequence data from type strains available through LPSN were downloaded, trimmed, and aligned to sequences from detected sequence variants. Species names were assigned to a sequence variant when only 16S rRNA gene fragments of previously described isolates of a single species were aligned with a maximum of two mismatches with this sequence variant. Relative abundances (in percentage) of sequence types, species, and genera were used for downstream analyses.

The analysis of extraction control samples had revealed the presence of slight but varying contamination levels in the FastDNASpinKit for soil, stably comprising *Ralstonia* sequence types, which may disturb the analysis in low biomass samples (Dos Anjos Borges et al. 2023). Additionally, the presence of bacteria of the *Enterococcus* and *Exiguobacterium* genera has been identified as contamination of paper points used for sampling subgingival communities (Szafranski et al. 2015; van der Horst et al. 2013). Correlation analysis was performed to identify potential contaminants in subgingival samples. Spearman correlations were calculated for species with a prevalence > 10% in subgingival samples and a network constructed based on pairwise Spearman correlations (*p* > 0.7) calculated in Prism 9 (GraphPad Software, Inc. Boston, MA, USA). The network was visualized using Cytoscape (version 3.9.1, San Diego, CA, USA). Three major cluster dominated by *Ralstonia solanacearum*, *Exiguobacterium aurantiacum*, and *Alkalibacterium pelagium/thalassium* were identified and sequence types of species exhibiting at least 3 strong correlations (*p* > 0.7) were removed (Supplementary Table S2b).

All samples were re-sampled before diversity analysis to equal the smallest library size of 20,714 reads using the phyloseq package. Calculation of diversity indices (species richness ST, Shannon diversity index H, Pielou`s evenness J) and multivariate analyses were performed using PRIMER (v.7.0.11, PRIMER-E, Plymouth Marine Laboratory, Plymouth, UK), whereas univariate analyses were performed using Prism 9. Differences in diversity indices were tested by ordinary ANOVA using the Holm-Sidak test for multiple comparisons.

The data matrices comprising sequence types, species, or genera (Supplementary Table S3a-d) were used to construct sample-similarity matrices applying the Bray–Curtis algorithm, where samples were ordinated using non-metric multidimensional scaling (nMDS) with 50 random restarts. Significant differences between a priori predefined groups of samples were evaluated using permutational multivariate analysis of variance (PERMANOVA), using type III (partial) sums of squares. Groups of samples were considered significantly different if the *p*-value was < 0.05. Centroids were calculated by PERMANOVA based on species level Bray–Curtis similarity matrices. The abundances of taxa present in the community of at least 10% of the samples were compared by the Friedman test in R and using the packages tidyverse, ggpubr, and rstatix. Benjamini–Hochberg corrections were applied for multiple comparisons (Hochberg and Benjamini 1990). Groups of samples were considered significantly different if the adjusted *p*-value was < 0.05. Taxa differentially distributed over time with an adjusted *p*-value (*q*) of < 0.1 were further assessed by Dunn’s post-hoc test. A two-way repeated measures ANOVA of square root transformed t0 and t1 relative abundance data to normalize skewed distributions was used to assess the effect of juice consumption on microbial communities. Statistically significant pairwise differences were corrected by the Sidak post-hoc test. An effect of juice consumption was considered when a significant interaction between the effects of the factors time and juice consumption was determined (*p* < 0.05). Only taxa where no significant difference in relative abundance at t0 between controls and juice consumers was observed were further analyzed. An effect of juice consumption was considered as probable when the mean abundance of a taxon differed significantly in either juice consumers or controls, but no difference was indicated (*p* > 0.1) in the respective other group. It was also considered as probable when the mean abundance differed significantly at time 1 but not at time 0 (*p* > 0.1). The within-group homogeneity was tested by calculating multivariate dispersion indices with PRIMER.

## Results

In the current study, oral samples were taken from 22 volunteers (Supplementary Table S1) at three different locations (saliva, dorsum of the tongue, and subgingival plaque). All volunteers were sampled before and 2 weeks as well as 6 months after placement of fixed orthodontic appliances giving 198 samples. Eleven of the volunteers received beet juice for 2 weeks after orthodontic treatment. Illumina sequencing of amplicons of the V1-V2 variable region of the 16S rDNA gave a total of 11,183,949 bacterial 16S rDNA sequence counts with a mean of 56,486 ± 20,725 reads per sample (Supplementary Table S2a). Of these, 10,938,972 bacterial 16S rDNA sequence counts remained after elimination of contaminations (see Materials and Methods) with a mean of 55,247 ± 21,383 reads per sample (Supplementary Table S2b).

### Determination of gingival indexes

After the insertion of fixed orthodontic appliances, typical gingival inflammatory parameters were determined. Within 2 weeks (t1), an increase in plaque accumulation (PI) and bleeding on probing (PBI) was indicated in both the control as well as the juice consuming group (Table 1). Notably, there was a significant difference in PBI between the control and juice consuming groups at t1 which remained even at t2 (Table 1). Moreover, the PI showed a significant increase from t0 to t2 (*p* = 0.005) in the control group, whereas such an increase was less pronounced in juice consumers. There was no change in the PSI and thus no development of periodontal diseases according to pocket depth of ≤ 3.4 mm (Score 1–2) from t0 to t2 (Table 1).

**Table 1:** Gingival conditions as assessed by Plaque Index, Papilla Bleeding Index, and Periodontal Screening Index. Gingival conditions of participants were assessed at three time points during this study: t0 (approximately 1 week before placement of the fixed orthodontic appliances), t1 (2 weeks after placement), and t2 (6 months after placement). Eleven participants received daily 120 mL of beet juice after placement (juice group), and eleven further participants served as controls (control group). Changes in gingival conditions were assessed by two-way repeated measures ANOVA and a mixed effects model in case of missing data. Results of multiple comparisons are shown where the Sidak test was used for correction. Significant *p*-values (*p*<0.05) are shown in bold

		Index ± SD or significance of difference between groups			Significance of difference in index size between groups analyzed at indicated times		
Index considered	Group analyzed	t0	t1	t2	t0, t1	t1, t2	t0, t2
Plaque Index (Score 0–4)	Control	0.7 ± 0.4	0.9 ± 0.5	1.2 ± 0.8	*p* = 0.346	*p* = 0.429	***p* = 0.005**
	Juice	0.3 ± 0.4	0.6 ± 0.4	0.7 ± 0.6	*p* = 0.526	*p* = 0.977	*p* = 0.138*
	Control vs. Juice	*p* = 0.533	*p* = 0.429	*p* = 0.149			
Papilla Bleeding Index (%)	Control	39.3 ± 4.3	59.4 ± 5.7	55.1 ± 6.0	*p* = 0.060	*p* > 0.999	*p* = 0.165
	Juice	22.7 ± 8.5	26.9 ± 8.0	25.6 ± 7.0	*p* = 0.994	*p* > 0.999	*p* = 0.999
	Control vs. Juice	*p* = 0.305	***p* = 0.008**	***p* = 0.014**			
Periodontal Screening Index (Score 0–4)	Control	1.0 ± 0.5	1.4 ± 0.5	1.3 ± 0.6	*p* = 0.564	*p* = 0.999	*p* = 0.307
	Juice	0.8 ± 0.4	1.0 ± 0.5	1.1 ± 0.4	*p* > 0.999	*p* > 0.999	*p* > 0.999
	Control vs. Juice	*p* > 0.999	*p* = 0.611	*p* = 0.433			

^*^An uncorrected *p*-value of 0.024 was determined

### Community differences between sampling sites

PERMANOVA analysis revealed significant community structure differences from sequence type to the genus level between the three distinct sampling sites (saliva, SA; dorsum of the tongue, TO; and subgingival plaque, SU) (Supplementary Table S4).

**Fig. 1:**
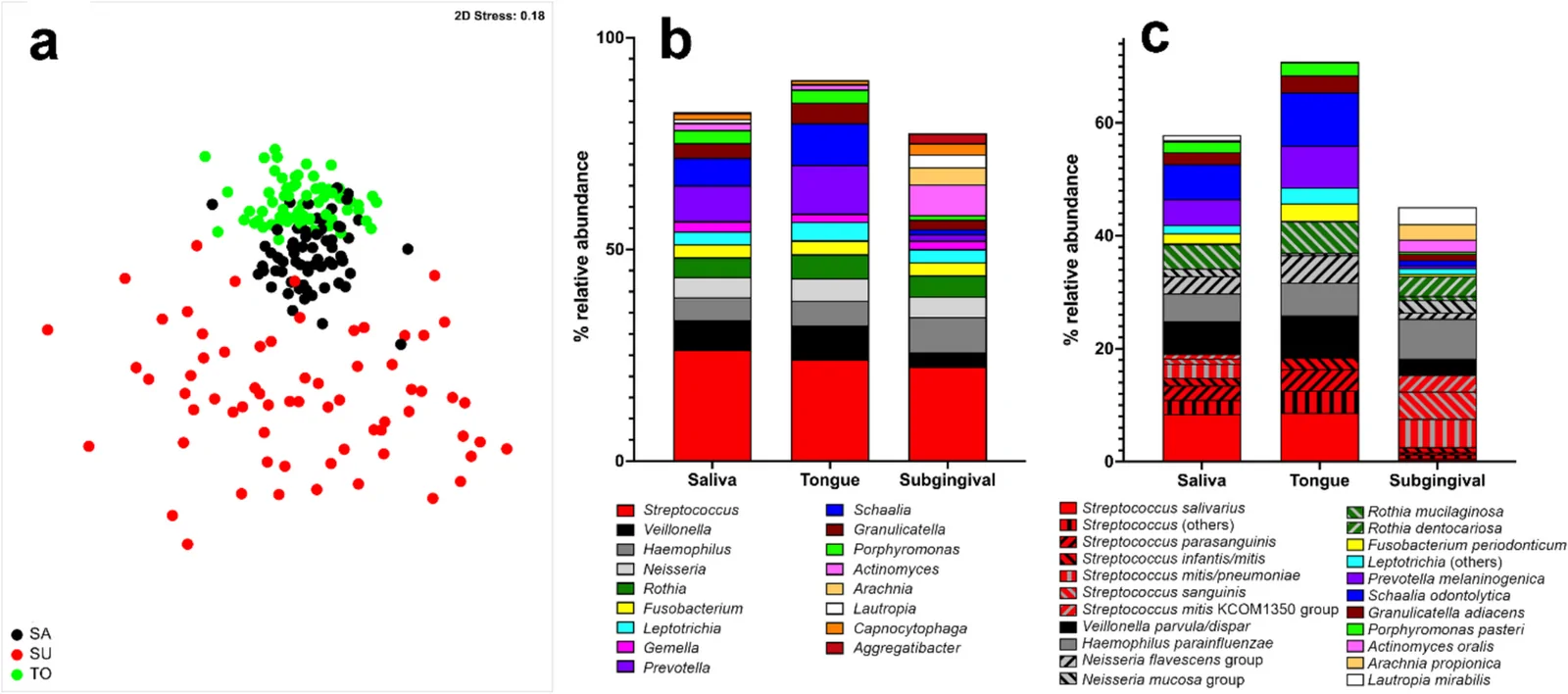
Differences in bacterial community structure in saliva, SA; dorsum of the tongue, TO; and subgingival plaque, SU. **a** The global bacterial community structure was assessed by non-metric multidimensional scaling (nMDS) and is based on standardized species abundance data. Similarities were calculated using the Bray–Curtis similarity algorithm. **b** Relative abundance of genera predominant in saliva, tongue, and subgingival plaque samples. Only genera with a mean relative abundance > 3% on at least one site are shown. **c** Relative abundance of species predominant in saliva, tongue, and subgingival plaque samples. Only species with a mean relative abundance > 2% on at least one site are shown. Sequence variants which could not be assigned to a species are joined as “others”

These differences were also visible in the nMDS plot with specifically SU communities being very different to SA and TO communities (Fig. 1a). Based on the Bray–Curtis resemblance matrix, dissimilarities of 53% and 42% between the centroids of TO and SA communities to SU communities could be calculated, whereas the dissimilarity between SA and TO communities was only 22%. Also, time and application of nitrate-containing juice had a significant influence on the global community structure.

Analysis of samples not subject to any treatment (t0) revealed a high abundance of *Schaalia* and *Prevotella* (specifically *Schaalia odontolytica* and *Prevotella melaninogenica*) in saliva and tongue samples, contrasting a high relative abundance of *Actinomyces, Arachnia*, and *Aggregatibacter* in subgingival samples (specifically *Actinomyces oralis* and *Arachnia propionica*) (Fig. 1b and c). However, even though similar amounts of *Streptococcus* sp. or *Rothia* sp. were present at the sites, the dominant species were different and *Rothia mucilaginosa* dominated in SA and TO, whereas *Rothia dentocariosa* was more abundant in SU. *Streptococcus salivarius* and *Streptococcus parasanguinis* dominated in SA and TO contrasting a high abundance of organisms related to *Streptococcus mitis* and *Streptococcus sanguinis* in SU.

### Community diversity at different sampling sites depending on carriage of orthodontic appliances and juice consumption

The differences in bacterial communities at the different regions were also visible in the diversity indices. SA showed the highest richness, diversity, and evenness, with the most significant differences to the other sampling sites being observed at the sequence type and species level. Differences between TO and SU communities were minor (Supplementary Fig. S1). Due to the high difference in community structure and diversity between the different regions, subsequent analyses were performed separately for the different regions.

Only minor effects were observed for the influence of time (placement of orthodontic appliances) and juice consumption (only between t0 and t1) on bacterial diversity. As an example, richness (Supplementary Fig. S2) increased slightly in TO samples from 50.5 ± 8.5 genera at t0 to 56.9 ± 8.6 genera at t2 in control and from 44.9 ± 6.1 genera at t0 to 50.4 ± 10.3 at t2 in juice consuming individuals. No significant effects were obvious in SA samples (Supplementary Fig. S2). Also, only minor changes were observed regarding Shannon diversity which, in contrast to the richness, decreased in TO samples over time (from 2.45 ± 0.23 at t0 to 2.07 ± 0.41 at t2 on the genus level in juice consuming individuals). Shannon diversity decreased also in SA samples (from 2.84 ± 0.47 at t0 to 2.51 ± 0.21 at the genus level at t2 in control and from 2.72 ± 0.42 at t0 to 2.43 ± 0.32 at t2 in juice consuming individuals, Supplementary Fig. S3). SU communities behaved differently, and an increasing diversity was detected in juice consuming patients (from 2.39 ± 0.47 at t0 to 2.74 ± 0.35 at t2). Results similar to those observed for Shannon diversity were revealed for Pielou’s evenness (Supplementary Fig. S4).

### Community structure at different sampling sites depending on carriage of orthodontic appliances and juice consumption

Two-way PERMANOVA analysis revealed the effect of orthodontic appliances (time) and juice consumption on SU, SA, and TO communities (Table 2). All three communities were significantly influenced by both time and juice on the species level, whereas analysis on the sequence type level analysis did not provide relevant information regarding community structure changes over time. Genus level analysis was less sensitive compared to species level analysis to reveal significant effects of juice application.

**Table 2:** Factors influencing SU sample community structures as indicated by PERMANOVA. The significance of differences in community structure of subgingival (SU), saliva (SA), and tongue (TO) samples at different times and depending on juice consumption was calculated by PERMANOVA (main test). The Pseudo-*F* and the *p*-values are given for each factor performed at different taxonomic levels (from sequence type to genus). The *t*-statistics and the *p*-values are also given for paired tests among different times as well as on subgroups of samples. Analysis was performed at different taxonomic levels (from sequence type to genus). Significant *p*-values *p* are shown in bold

Region		SU						SA						TO					
Factor analyzed		Sequence type		Species		Genus		Sequence type		Species		Genus		Sequence type		Species		Genus	
		Pseudo-*F*	*p*	Pseudo-*F*	*p*	Pseudo-*F*	*p*	Pseudo-*F*	*p*	Pseudo-*F*	*p*	Pseudo-*F*	*p*	Pseudo-*F*	*p*	Pseudo-*F*	*p*	Pseudo-*F*	*p*
Time		1.043	0.302	1.535	**0.017**	1.851	**0.020**	1.022	0.404	2.532	**0.002**	4.722	**0.001**	0.861	0.782	2.321	**0.006**	3.346	**0.001**
Juice		1.434	**0.009**	1.852	**0.010**	2.057	**0.031**	1.710	**0.003**	1.776	**0.041**	1.646	0.104	1.827	**0.009**	2.063	**0.040**	1.829	0.119
Time x juice		0.763	0.999	0.876	0.713	1.044	0.366	0.496	1.000	0.632	0.971	0.684	0.789	0.547	0.998	0.566	0.944	0.595	0.793
	Groups compared	*t*	*p*	*t*	*p*	*t*	*p*	*t*	*p*	*t*	*p*	*t*	*p*	*t*	*p*	*t*	*p*	t	*p*
All samples	t0, t1	1.122	**0.043**	1.443	**0.007**	1.670	**0.005**	0.814	0.984	0.988	0.478	0.947	0.511	0.688	0.997	0.975	0.426	1.082	0.287
	t0, t2	1.051	0.189	1.293	**0.016**	1.355	**0.045**	1.165	0.035	1.967	**0.001**	2.898	**0.001**	1.135	0.125	1.960	**0.003**	2.469	**0.002**
	t1, t2	0.873	0.979	0.895	0.714	0.896	0.603	1.032	0.337	1.721	**0.001**	2.461	**0.001**	0.911	0.716	1.492	**0.027**	1.724	**0.019**
Control samples (C)	t0, t1	1.042	0.193	1.280	0.061	1.251	0.121	0.780	0.980	0.989	0.457	0.905	0.628	0.644	0.993	0.843	0.659	0.967	0.406
	t0, t2	0.963	0.698	1.073	0.240	1.083	0.282	1.040	0.320	1.638	**0.001**	2.333	**0.001**	0.901	0.711	1.316	0.097	1.709	**0.039**
	t1, t2	0.862	0.974	0.931	0.620	0.846	0.678	0.947	0.699	1.314	**0.038**	1.841	**0.001**	0.783	0.943	0.927	0.503	1.052	0.305
Juice consumers (J)	t0, t1	0.981	0.578	1.181	0.114	1.455	**0.027**	0.686	0.994	0.837	0.769	0.892	0.582	0.706	0.992	0.897	0.555	0.914	0.503
	t0, t2	0.942	0.774	1.070	0.239	1.242	0.118	0.915	0.801	1.386	**0.042**	2.008	**0.002**	0.993	0.466	1.595	**0.023**	1.864	**0.007**
	t1, t2	0.898	0.903	1.003	0.424	1.251	0.109	0.818	0.946	1.314	0.060	1.786	**0.023**	0.949	0.609	1.408	0.046	1.594	**0.047**
Time 0	C, J	0.966	0.706	1.053	0.307	1.120	0.225	0.913	0.912	0.940	0.616	0.895	0.621	0.932	0.768	0.984	0.433	0.828	0.648
Time 1	C, J	1.084	0.072	1.357	**0.020**	1.455	0.054	1.011	0.412	1.152	0.166	1.121	0.241	1.081	0.188	1.318	0.090	1.307	0.126
Time 2	C, J	0.925	0.899	0.832	0.892	0.912	0.579	0.915	0.873	0.873	0.770	0.931	0.499	0.938	0.666	0.665	0.967	0.738	0.768

Significant *p*-values (p<0.05)

Interesting effects were observed in pairwise tests. SU communities (Table 2) showed pronounced community structure changes between t0 and t1 or t0 and t2, indicating a major influence on the community after placement of the fixed orthodontic appliances. Such a change in community composition between t0 and t1 was more pronounced in juice consumers, and communities of juice consuming patients were significantly different to communities of control patients only at t1.

In contrast to SU communities, time-dependent changes in SA and TO communities were visible mainly between t0 and t2 and between t1 and t2 (Table 2) and thus during the time with fixed orthodontic appliances in place. Community changes were observed in both, control and juice consuming patients, with no significant differences in communities between those groups at any time.

### Taxa differentially distributed depending on carriage of orthodontic appliances (time)

**Fig. 2:**
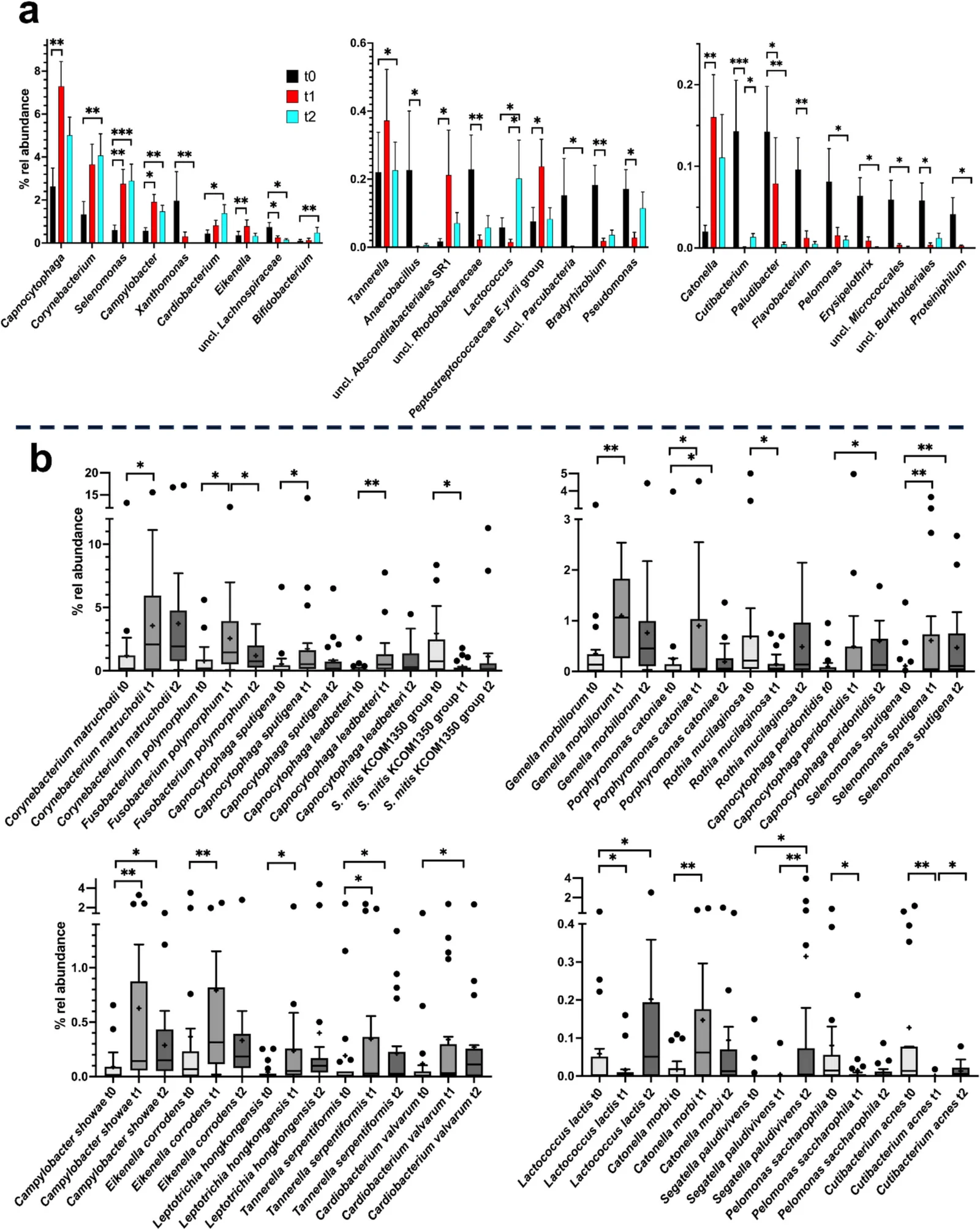
Relative mean abundance of genera and genus level taxa as well as of species in SU samples. **a** The mean relative abundance (% rel abundance) of genera and genus level taxa at time 0, 1, and 2 is displayed as well as the standard error of mean. Differences in taxon distribution were evaluated by the Friedman test. If a differential distribution was indicated (adjusted *p* < 0.05), taxa differentially distributed over time were further assessed by Dunn’s post-hoc test. Statistically significant differences are indicated as *p < 0.05, **p < 0.01, or ***p < 0.001. **b** The relative abundance of species is given at time 0, 1, and 2. Differences in taxon distribution were evaluated by the Friedman test. If a differential distribution was indicated, taxa differentially distributed over time were further assessed by Dunn’s post-hoc test. Statistically significant differences are indicated as *p < 0.05, **p < 0.01, or ***p < 0.001. The median is indicated by a black line and the mean by +. The box represents the interquartile range (IQR). The whiskers extend to the upper adjacent value (largest value = 75 th percentile + 1.5 × IQR) and the lower adjacent value (lowest value = 25 th percentile − 1.5 × IQR) and the dots represent outliers

Forty-eight of 179 genus level taxa present in at least 10% of SU samples were differentially distributed during time according to the Friedman test (adjusted *p*-value (*q*) < 0.05) with 27 taxa showing differences in distribution in a pairwise test (Supplementary Table S5). As indicated by PERMANOVA, a remarkable change occurred between t0 and t1 with 17 genera being differentially distributed among these time points but only 2 between t1 and t2 (Fig. 2a). Most prominently, *Campylobacter* increased from a mean relative abundance of 0.57% to an abundance of 1.91%, *Capnocytophaga* from 2.63 to 7.29%, and *Selenomonas* from 0.60 to 2.75%. Typically, the high relative abundance remained during the further course of the experiment. Various genera decreased in abundance such as *Xanthomonas* or *Anaerobacillus. Lactococcus* increased in abundance specifically at t2.

Analysis on species level confirmed above results on time-dependent community changes in SU samples and revealed *Campylobacter showae*, *Capnocytophaga leadbetteri*, *Capnocytophaga sputigena*, *Catonella morbi*, *Corynebacterium matruchotii*, *Eikenella corrodens*, *Fusobacterium polymorphum*, *Gemella morbillorum*, *Leptotrichia hongkongensis*, *Porphyromonas catoniae*, *Selenomonas sputigena*, and *Tannerella serpentiformis* to be all enriched after application of the fixed orthodontic appliances, whereas *Cutibacterium acnes, Lactococcus lactis*, *Pelomonas saccharophila*, *R. mucilaginosa*, and organisms related to *S. mitis* KCOM350 were depleted (Fig. 2). However, some of these species (*C. showae*, *C. leadbetteri*,* C. sputigena*,* C. morbi*,* C. matruchotii*, *E. corrodens*) may be influenced by juice consumption rather than application of orthodontic appliances (see below).

Twenty-one of 142 genus level taxa present in at least 10% of SA samples were indicated to be differentially distributed (Friedman test, adjusted *p*-value (*q*) < 0.05) with 13 showing differences in distribution in a pairwise test (Supplementary Table S5). Ten further genera were indicated to be differentially distributed when the Friedman test with *p* < 0.05 and 0.05 < *q* < 0.1 was applied but showed a significant differential distribution in the pairwise test.

**Fig. 3:**
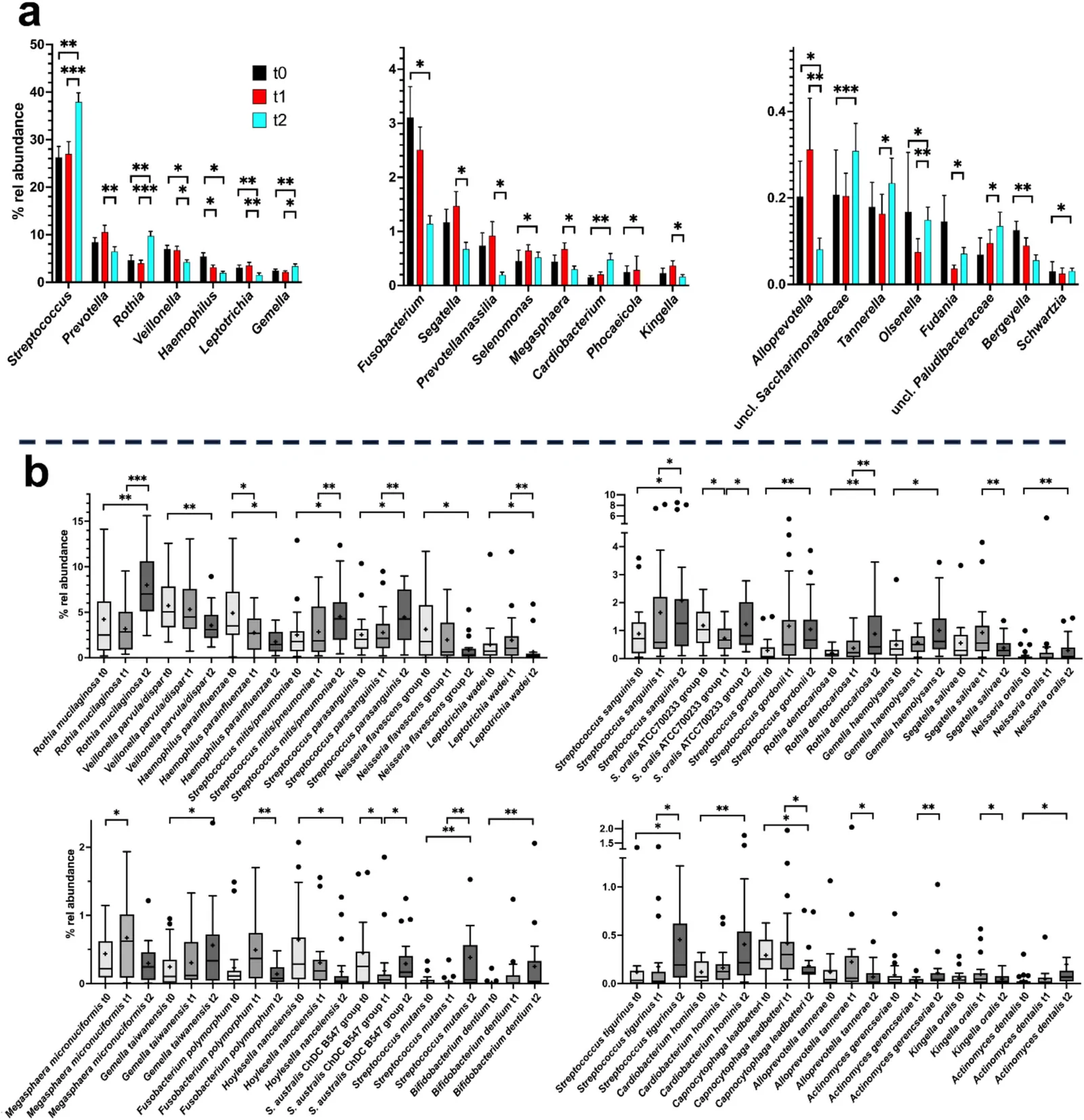
Relative mean abundance of genera and genus level taxa as well as of species in SA samples. **a** The mean relative abundance (% rel abundance) of genera and genus level taxa at time 0, 1, and 2 is displayed as well as the standard error of mean. Differences in taxon distribution were evaluated by the Friedman test. If a differential distribution was indicated (adjusted *p* < 0.05), taxa differentially distributed over time were further assessed by Dunn’s post-hoc test. Statistically significant differences are indicated as *p < 0.05, **p < 0.01, or ***p < 0.001. **b** The relative abundance of species is given at time 0, 1, and 2. Differences in taxon distribution were evaluated by the Friedman test. If a differential distribution was indicated, taxa differentially distributed over time were further assessed by Dunn’s post-hoc test. Statistically significant differences are indicated as *p < 0.05, **p < 0.01, or ***p < 0.001. The median is indicated by a black line and the mean by +. The box represents the IQR. The whiskers extend to the upper adjacent value (largest value = 75 th percentile + 1.5 × IQR) and the lower adjacent value (lowest value = 25 th percentile − 1.5 × IQR), and the dots represent outliers

In contrast to SU samples, where community changes were observed mainly between t0 and t1, the most obvious changes in SA communities occurred between t1 and t2 with 15 genera being significantly differentially distributed between these time points but only 1 between t0 and t1 (Fig. 3). In accordance with the different SU and SA community compositions, only *Cardiobacterium* and *Selenomonas* reacted similarly to the application of orthodontic appliances in both communities.

Importantly, *Streptococcus,* the most abundant genus in SA increased from a mean relative abundance of 27.0 ± 2.6% at t1 to an abundance of 37.9 ± 1.9% at t2 and the relative abundance of *Rothia* more than doubled from 4.02 ± 0.61% to 9.73 ± 0.98%. There was no change in abundance after placement of orthodontic appliances at t1 compared to t0. An increase in relative abundance was also evident for *Gemella* and *Tannerella*, whereas *Prevotella*, *Veillonell*a, *Leptotrichia*, and *Fusobacterium* among other declined in relative abundance. A significant relative abundance change between t0 and t1 was only obvious for the decline of *Haemophilus*.

Analysis on the species level gave more information on the taxa subject to changes in relative abundance over time. As for the genera, most prominent changes were observed between t0 and t2 with different species reacting in SU and SA. As indicated from genus level analysis, different *Streptococcus* species (*S. mutans*, *S. parasanguinis*, *S. gordonii*, *S. tigurinus*, and Streptococci related to *S. mitis/pneumoniae*) and *Rothia* species (specifically *R. mucilaginosa* but also *R. dentocariosa*) were enriched over time in SA. However, some *Streptococcus* species showed a different behavior (Fig. 3). Also, *Actinomyces dentalis*, *Bifidobacterium dentium*, *Gemella haemolysans*, and *Cardiobacterium hominis* significantly increased in relative abundance, whereas *Haemophilus parainfluenza* or *Veillonella parvula/dispar* were depleted. Only *F. polymorphum* and *C. leadbetteri* changed significantly in relative abundance at both sites, and whereas the former always increased in abundance after placement of orthodontic appliances, *C. leadbetteri* increased immediately after placement in subgingival plaque but not in saliva.

**Fig. 4:**
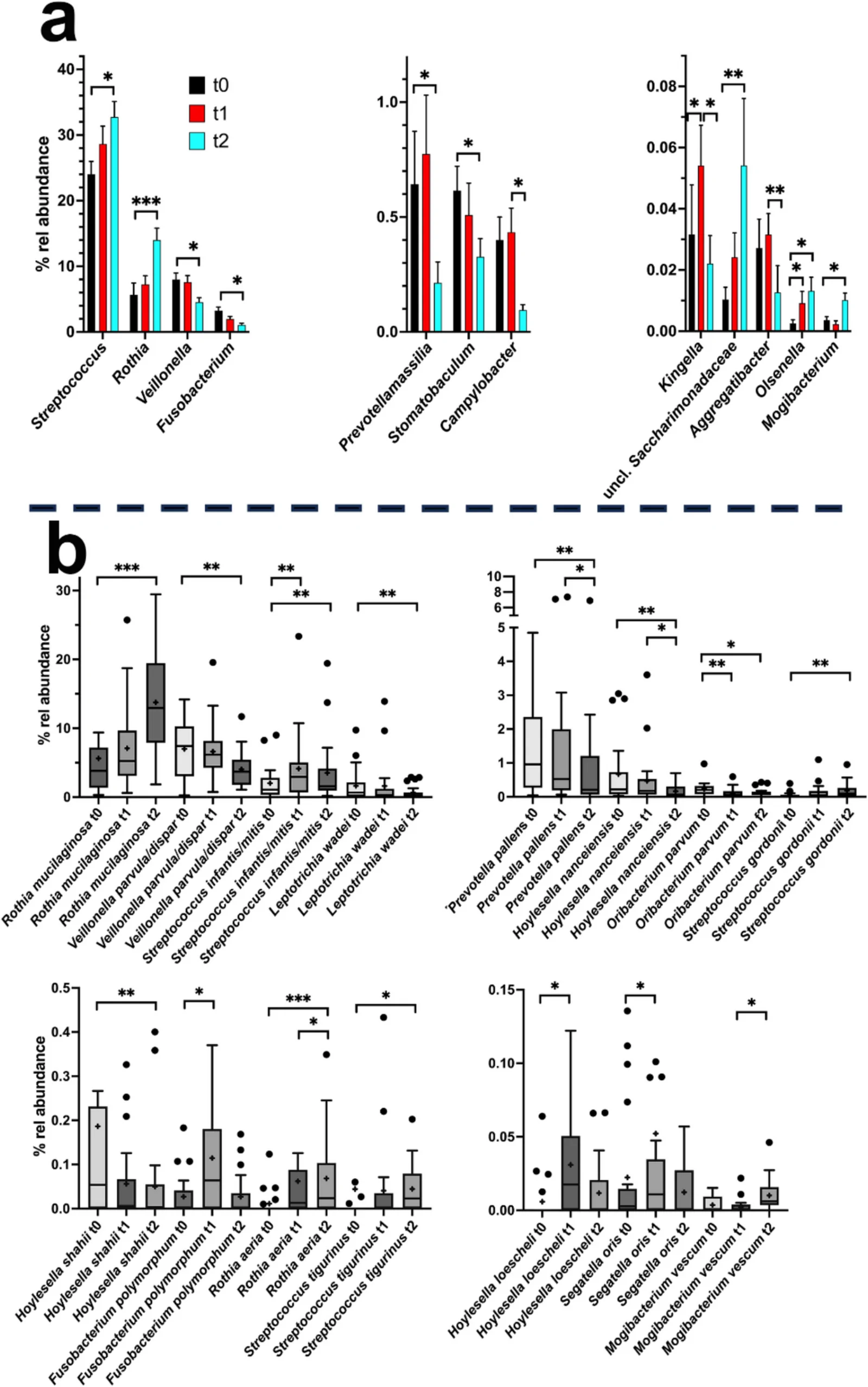
Relative mean abundance of genera and genus level taxa as well as of species in TO samples. **a** The mean relative abundance (% rel abundance) of genera and genus level taxa at time 0, 1, and 2 is displayed as well as the standard error of mean. Differences in taxon distribution were evaluated by the Friedman test. If a differential distribution was indicated (adjusted *p* < 0.05), taxa differentially distributed over time were further assessed by Dunn’s post-hoc test. Statistically significant differences are indicated as *p < 0.05, **p < 0.01, or ***p < 0.001. **b** The relative abundance of species is given at time 0, 1, and 2. Differences in taxon distribution were evaluated by the Friedman test. If a differential distribution was indicated, taxa differentially distributed over time were further assessed by Dunn’s post-hoc test. Statistically significant differences are indicated as *p < 0.05, **p < 0.01, or ***p < 0.001. The median is indicated by a black line and the mean by +. The box represents the IQR. The whiskers extend to the upper adjacent value (largest value = 75 th percentile + 1.5 × IQR) and the lower adjacent value (lowest value = 25 th percentile − 1.5 × IQR), and the dots represent outliers

Only 6 of 80 genus level taxa present in at least 10% of TO samples (7.5%) were differentially distributed (Friedman test, adjusted *p*-value (*q*) < 0.05), much less compared to SU (26.8%) or SA samples (14.8%) (Supplementary Table S5). Five of those genera showed differences in distribution in a pairwise test. Seven further genera were indicated to be differentially distributed when the Friedman test with *p* < 0.05 and 0.05 < *q* < 0.1 was applied but showed a significant differential distribution in the pairwise test. As for SA samples, the clearest community changes were observed between t0 and t2 (Fig. 4), and only *Kingella* was differentially distributed between t0 and t1.

Generally, changes were similar to those observed in SA communities, with *Streptococcus*, *Rothia*, and unclassified *Saccharimonadaceae* being enriched and *Veillonella*, *Fusobacterium*, and *Prevotellamassilia* being depleted. *Kingella* increased in abundance between t0 and t1 after placement of the orthodontic appliances but showed a subsequent depletion to reach t0 levels.

Also on the species level, changes were similar to those observed in SA samples and as an example, *R. mucilaginosa* increased in abundance over time, whereas *F. polymorphum* obviously peaked after placement of orthodontic appliances (as observed for this species also in SU samples). However, less species were differentially distributed in TO samples depending on carriage of fixed orthodontic appliances.

### Taxa differentially distributed depending on juice consumption

**Fig. 5:**
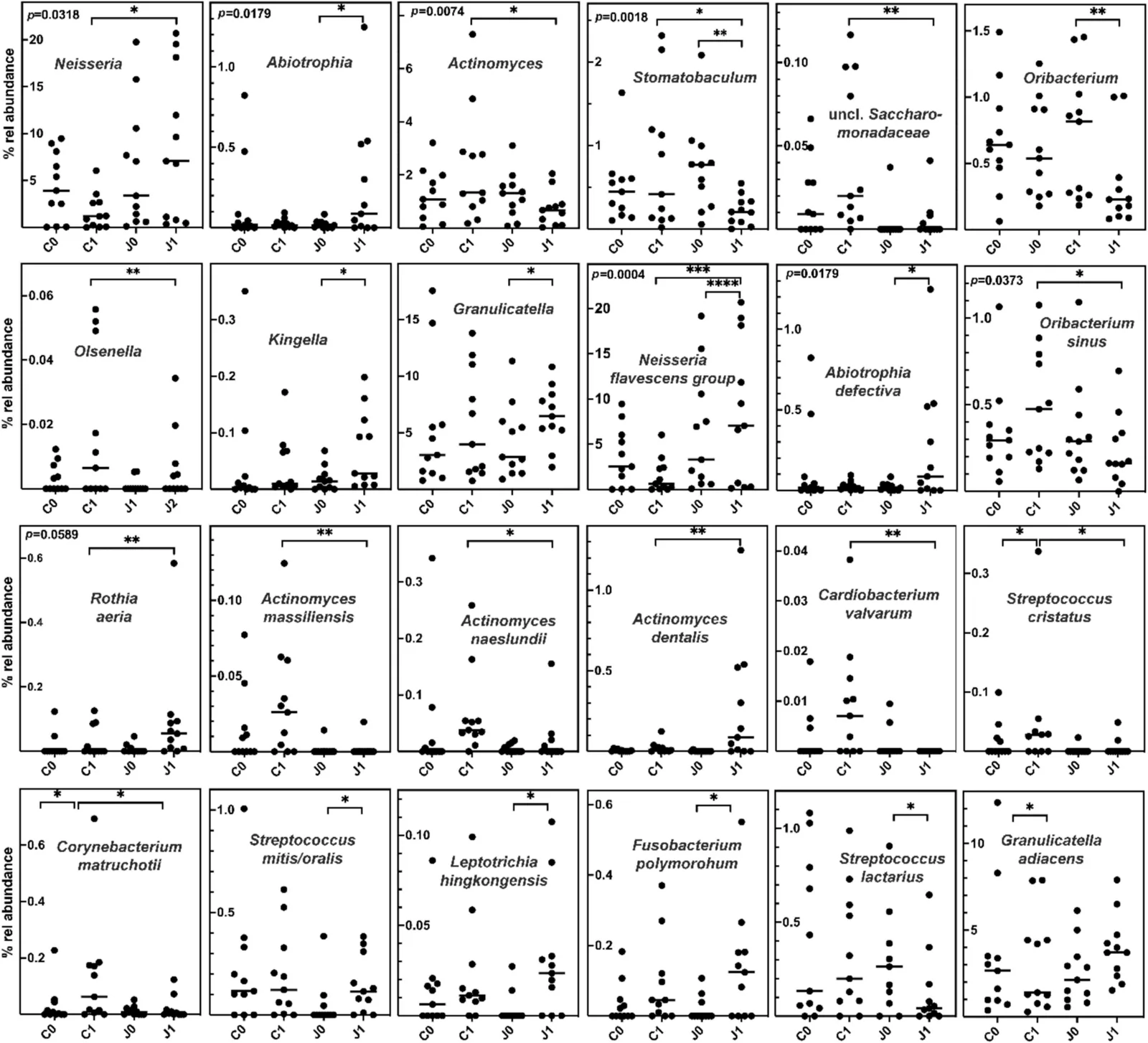
The effect of juice on the relative abundance of selected taxa in TO samples. The relative abundance (% rel abundance) at time 0 and 1 and separately in controls (C) or juice consuming patients (J) is displayed as well as the median. Differences in taxon distribution were evaluated by a 2-way repeated measures ANOVA on square root transformed abundance data. Statistically significant pairwise differences were corrected by the Sidak post-hoc test and are indicated as *p < 0.05, **p < 0.01, ***p < 0.001, and ****p < 0.0001. The significance of interaction between the factors time and juice consumption is indicated as insert to each graph. Untransformed relative abundance data are visualized

Out of the 22 patients analyzed, eleven received a nitrate-containing juice for a period of 2 weeks. Thus, a difference in communities based on juice consumption should be visible just after 2 weeks but not in t0 samples, nor in t2 samples. Analysis was therefore based on a two-way ANOVA of square root transformed t0 and t1 relative abundance data to normalize skewed distributions (Supplementary Table S6). The effect of juice consumption on tongue microbial communities was evident for *Neisseria* and *Abiotrophia* as well as for *Actinomyces* and *Stomatobaculum* where a significant interaction between the factors time and juice consumption was determined and relative abundances were affected (as shown by the post-hoc test) only at t1 but not at t0. Clearly, juice consumption increased the abundance of *Neisseria* and *Abiotrophia* but resulted in a depletion of *Actinomyces* and *Stomatobaculum.* Neither the relative abundance of *Rothia* nor that of *Veillonella* were affected by juice consumption. Based on their significant difference in relative abundance after juice consumption compared to the control and/or an increase compared to the t0 level, *Kingella* and *Granulicatella* are probably enriched and *Oribacterium* depleted, whereas the enrichment of unclassified *Saccharimonadaceae* and *Olsenella* is probably prevented (Fig. 5 and Supplementary Table S6).

Analysis on the species level revealed that enrichment occurred for *Abiotrophia defectiva* and organisms of the *Neisseria flavescens* group, whereas no effect was observed on organisms of the *Neisseria mucosa* group or *Neisseria oralis.* Out of the *Rothia* species, only *Rothia aeria* tended to be enriched, an effect which was masked on the genus level by the absence of an influence on *R. mucilaginosa* or *R. denticariosa. Oribacterium sinus* was clearly depleted. For different *Actinomyces* species (*A. massiliensis*, *A. naeslundii*, *A. dentalis*) as well as for *C. matruchotii*, *Streptococcus cristatus*, and *Cardiobacterium valvarum*, a time-dependent enrichment was evidently prevented by juice consumption (Fig. 5).

**Fig. 6:**
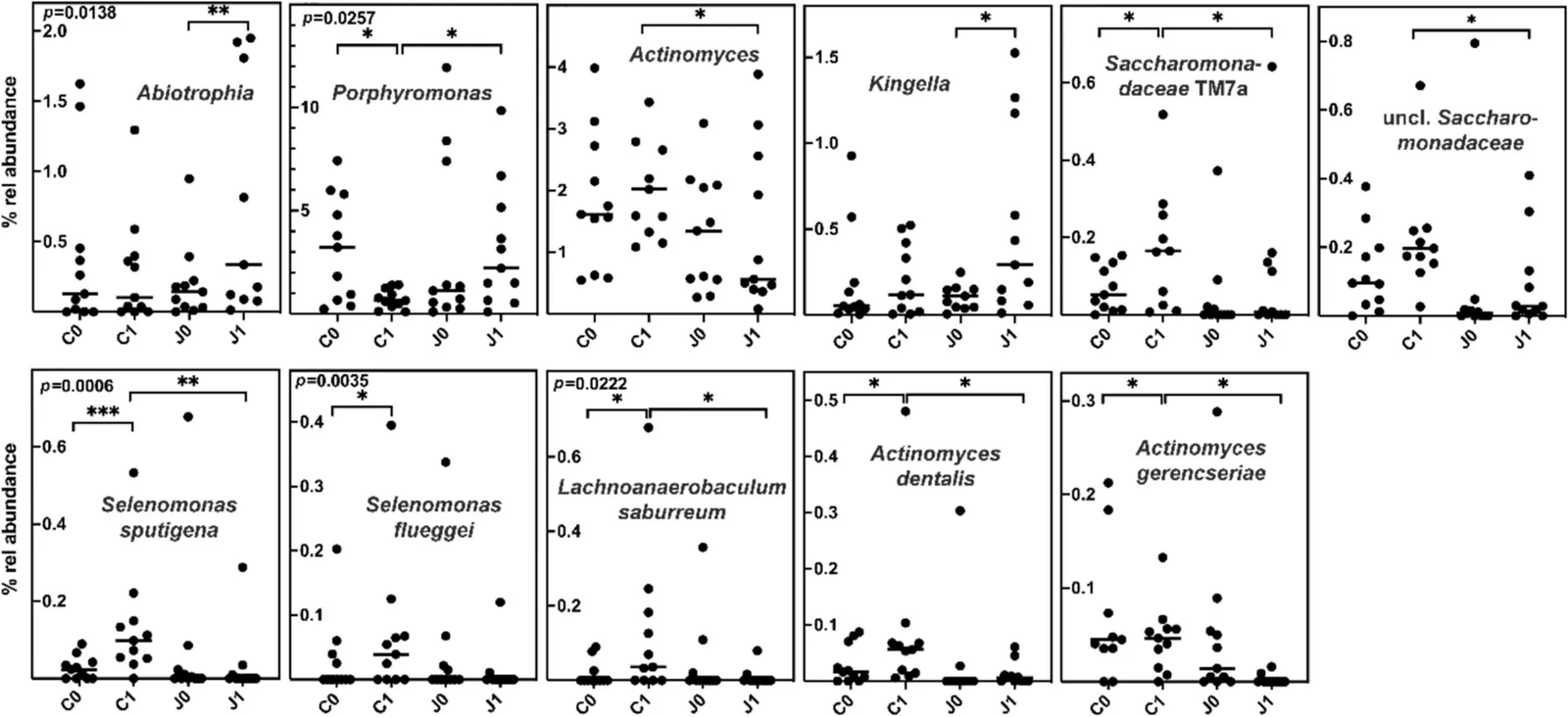
The effect of juice on the relative abundance of selected taxa in SA samples. The relative abundance (% rel abundance) at time 0 and 1 and separately in controls (C) or juice consuming patients (J) is displayed as well as the median. Differences in taxon distribution were evaluated by a 2-way repeated measures ANOVA on square root transformed abundance data. Statistically significant pairwise differences were corrected by the Sidak post-hoc test and are indicated as *p < 0.05, **p < 0.01, and ***p < 0.001. The significance of interaction between the factors time and juice consumption is indicated as insert to each graph. Untransformed relative abundance data are visualized

As indicated by the PERMANOVA analysis, the influence of juice on the bacterial SA communities was also minor. Two-way ANOVA analysis of square root transformed t0 and t1 relative abundance data revealed only the *Abiotrophia* and *Porphyromonas* genera to exhibit a significant interaction between the factors time and juice consumption. As in TO communities, juice consumption obviously results in an enrichment of *Abiotrophia*. Whereas *Actinomyces* was depleted as in TO communities, no effect on the abundance of *Neisseria* or *Stomatobaculum* by juice consumption was observed in SA communities. As in TO communities, *Kingella* was probably enriched, and the enrichment of unclassified *Saccharimonadaceae* was probably prevented (Fig. 6).

On the species level, a time-dependent enrichment of *Actinomyces* species was prevented, as already shown for TO communities, but effects on *Rothia* or *Neisseria* species were absent. However, a prevention of a time-dependent enrichment could clearly be shown for *S. sputigena*, *Selenomonas flueggei*, and *Lachnoanaerobaculum saburreum* by two-way ANOVA (Fig. 6).

**Fig. 7:**
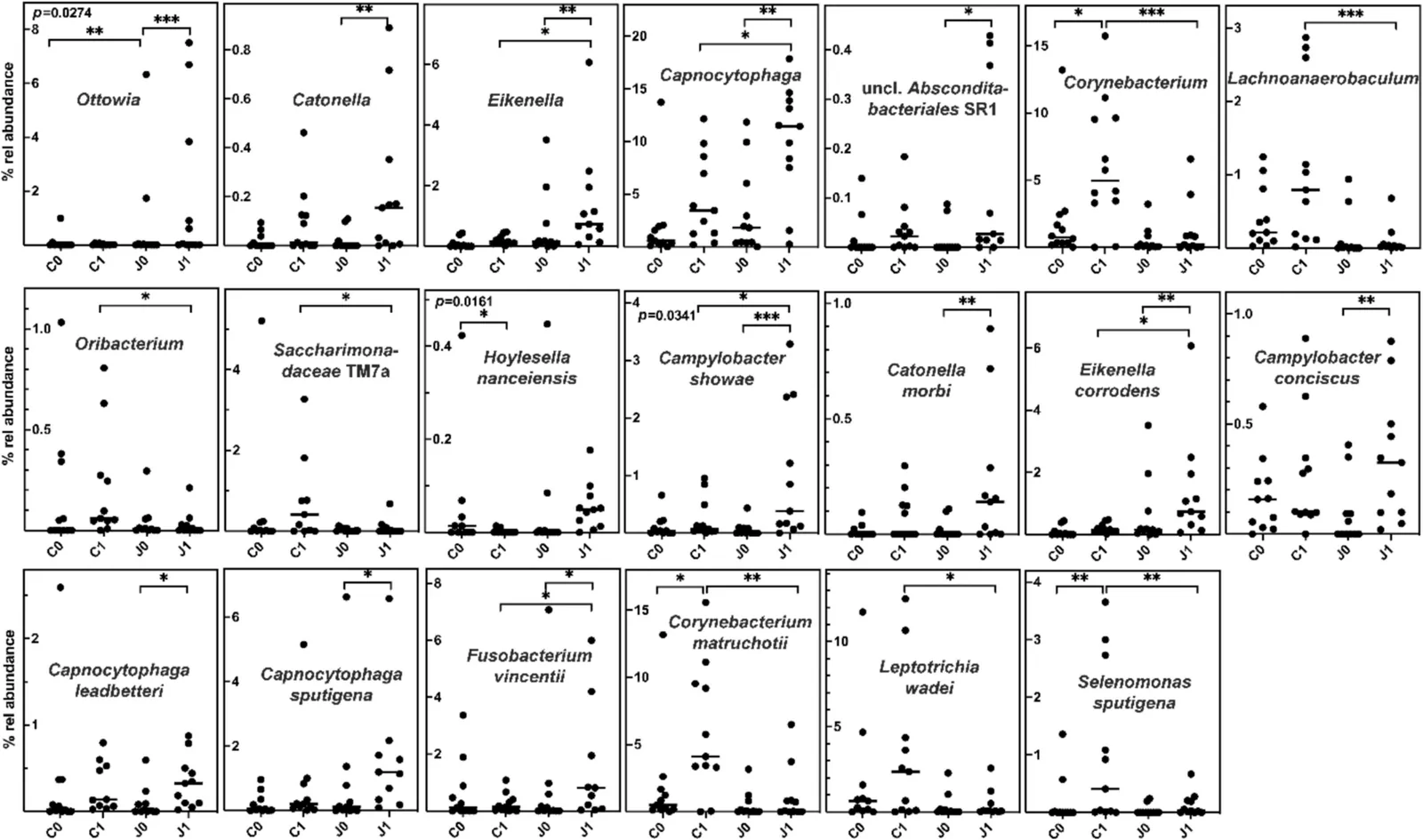
The effect of juice on the relative abundance of selected taxa in SU samples. The relative abundance (% rel abundance) at time 0 and 1 and separately in controls (C) or juice consuming patients (J) is displayed as well as the median. Differences in taxon distribution were evaluated by a 2-way repeated measures ANOVA on square root transformed abundance data. Statistically significant pairwise differences were corrected by the Sidak post-hoc test and are indicated as *p < 0.05, **p < 0.01, and ***p < 0.001. The significance of interaction between the factors time and juice consumption is indicated as insert to each graph. Untransformed relative abundance data are visualized

PERMANOVA analysis (Table 1) had indicated some influence of juice on the bacterial SU communities where significant differences were observed when communities collected after 2 weeks of juice consumption had been compared to controls on the species level (*p* = 0.02). The two-way ANOVA analysis of square root transformed t0 and t1 relative abundance data revealed only the *Ottowia* genus and the *Hoylesella nanceiensis* and *C. showae* species to exhibit a significant interaction between the factors time and juice consumption with all of them being enriched upon juice consumption (Fig. 7). Based on their significant increase in abundance after juice consumption compared to the control, *Eikenella* (and the species *E. corrodens*), *Capnocytophaga* (and the species *C. leadbetteri* and *C. sputigena*), *Catonella* (and the species *C. morbi*) as well as unclassified *Absconditabacteriales* (SR1) and *Campylobacter concisus* and *Fusobacterium vincentii* can also be assumed to be enriched by nitrate-containing juice. In contrast, juice consumption obviously prevented the enrichment of *Corynebacterium* (and the species *C. matruchotii*), *Lachnoanaerobaculum*, *Saccharimonadaceae* TM7a, and *Oribacterium* as well as the species *S. sputigena* and *Leptotrichia wadei* as indicated by an increase in abundance compared to the t0 level only in the control group (Fig. 7).

## Discussion

The human oral cavity hosts complex microbial communities which colonize not only the tooth surface but also the tongue, mucosa, hard palate, and the subgingival plaque. Orthodontic treatment can be associated with adverse effects, such as increased biofilm formation, change in composition of plaque, and difficulty in oral hygiene maintenance (Müller et al. 2021). The resulting imbalance of the oral microbiota increases the risk for enamel demineralization, dental caries, and periodontal disease (Kilian et al. 2016). This dysbiosis of the microbiota can be overcome by good oral hygiene and a balanced diet. However, despite the severe differences in oral communities, no study has compared the influence of orthodontic devices on these distinct communities nor was the influence of diet, specifically of a nitrate-containing diet during orthodontic treatment analyzed, yet.

As previously described, the microbial community at the tongue dorsum is tremendously different from that of the subgingival plaque (Baker et al. 2024) with the saliva community being similar to the tongue but also sharing some taxa with the subgingival plaque. This is assumed to be due to the fact that microorganisms from all oral sites are released into saliva (Mark Welch et al. 2019). However, distinct genera are specialized for diverse oral niches (such as *Actinomyces* for the subgingival plaque and *Schaalia* for the tongue). Additionally, distinct species from various genera were also described as specialists for the tongue versus the subgingival plaque such as microorganisms of the *N. flavescens* group versus those of the *N. mucosa* group, *R. mucilaginosa* versus *R. denticariosa* or *S. salivarius*, and *S. parasanguinis* versus *S. sanguinis* (Mark Welch et al. 2019; McLean et al. 2022). Thus, the effect of orthodontic treatment and nitrate consumption clearly needs to be addressed separately for different sites.

The necessity to separately analyze different sites is already evident from PERMANOVA analyses of the three environments, where subgingival communities reacted promptly to orthodontic treatment with no further overall structure change during extended treatment. In contrast, tongue but also saliva communities did not react during the first 2 weeks of placement but during further treatment up to 6 months (Table 2). Moreover, a significant influence of juice consumption was evident only during species level analysis of subgingival samples, whereas saliva samples were the least discriminative to identify an effect of nitrate-containing juice on overall communities.

The effect of orthodontic treatment on microbial communities has been analyzed by various authors typically either by plating or qPCR to identify the prevalence and abundance of a limited number of periodontal pathogens such as *Aggregatibacter actinomycetemcomitans*, *Porphyromonas gingivalis*, *Prevotella intermedia*, or *Tannerella forsythia* usually in subgingival samples (Guo et al. 2017; Kim et al. 2012), which all in the current report were in a prevalence too low for any statistical analysis. In recent years, high-throughput sequencing of part of the 16S rRNA gene or shotgun metagenomic sequencing has become popular allowing for a broad overview of the total community during orthodontic treatment, however, at a certain threshold abundance. Results of different studies appear somewhat contradictory, which may be due to different methods used (see above), different duration of orthodontic treatment, different niches sampled, or even different statistical methods used which may severely influence the outcome (Nearing et al. 2022).

High-throughput sequencing-based microbiota analysis after orthodontic treatment has been predominantly performed in subgingival samples (Babikow et al. 2024; Chen et al. 2022; Guo et al. 2019; Kado et al. 2020; Koopman et al. 2015). It has been reported that periodontal pathogens such as *Selenomonas* and *Porphyromonas* gained abundance during orthodontic treatment, while health associated *Streptococcus*, *Rothia*, and *Haemophilus* gained abundance towards the end and after orthodontic treatment (Koopman et al. 2015). However, analyses were typically done earliest 1 month after insertion of fixed orthodontic appliances (Guo et al. 2019) and up to 2 years (Koopman et al. 2015). Overall, most reports agree on the enrichment of periodontal pathogens, specifically *S. sputigena*, in accordance with our results. Importantly, as shown here, such community shift is already pronounced after 2 weeks, additionally reflected by the PBI which was specifically enhanced in the control group compared to the juice consuming group at t1 and t2 (*p* < 0.05) (Table 1). Also, the PI increased significantly between t0 and t2, however, again specifically in the control group. Though, there was no increase in periodontal sulcus depth (≤ 3.4 mm) over time according to the definition of PSI Scores (Charles and Charles 1994) which is therefore defined as gingivitis and not as periodontal disease (Score 1–2) (Szafranski et al. 2015). The observations are in agreement with other reports that discuss the general effects of orthodontic treatment on the plaque amount and periodontium (Alexander 1991; Reichardt et al. 2019). Such alterations occurred despite repeated motivation and awareness of oral hygiene techniques before and during treatment and showed a decrease in motivation over time and difficulties in brushing properly the fixed appliances (Zachrisson and Zachrisson 1971).

Saliva is a second niche where the effect of orthodontic treatments has been assessed previously (Kado et al. 2020; Wang et al. 2024; Zhao et al. 2020). Importantly, whereas a decrease in abundance of periodontal pathogens such as *Porphyromonas* and *Prevotella* and an increase in *Neisseria* has been reported (Zhao et al. 2020), also, completely opposite results have been published (Kado et al. 2020). The last report, who analyzed both saliva and subgingival plaque, strangely, did not observe differences and claimed that results in saliva just reflect the results seen in subgingival biofilm, despite the enormous community differences reported (Baker et al. 2024). Interestingly, effects on *Streptococcus* species were only seldom indicated. We could show here a significant increase in relative abundance of the genus overall but also succeeded in showing an increase in abundance of the pathogenic species *S. mutans* in saliva in accordance with previous qPCR results (Guo et al. 2019). Analysis of the effect of orthodontic treatment on tongue communities has, to our knowledge, not previously been reported. As expected from communities relatively similar to those of the saliva, results to a certain extend mirror those observed in saliva with *Streptococcus*, *Rothia*, and *Saccharimonadaceae* being enriched. Specifically, the species *R. mucilaginosa*, described as specialized for the tongue niche (Mark Welch et al. 2019), increased tremendously in abundance in both tongue and saliva communities.

Previous reports have claimed that regular nitrate administration results in higher relative abundances of the oral health-associated nitrate-reducing genera *Neisseria* and *Rothia*, a depletion of caries-associated genera such as *Veillonella* or *Leptotrichia* (Rosier et al. 2022), and that nitric oxide production is responsible for limiting inflammation (Jockel-Schneider et al. 2021). Therefore, nitrate intake is proposed as an additional therapeutic measurement to maintain good oral hygiene in patients with higher risks for caries and periodontitis such as the case for orthodontic treatment. Thus, the selective advantage of denitrifiers such as *Neisseria* and *Rothia* (Jockel-Schneider et al. 2021; Vanhatalo et al. 2018) gain in the presence of nitrate could further benefit the gingiva. This is documented here by a reduction of bleeding on probing after 2 weeks of nitrate intake in the juice consuming group (Table 1). Previous analyses of communities affected by nitrate intake have been performed by high-throughput sequencing in saliva both in vivo (du Toit et al. 2024; Rosier et al. 2021; Vanhatalo et al. 2018; Velmurugan et al. 2016) and in vitro (Rosier et al. 2020) and gave evidence for enrichment of *R. mucilaginosa* or *N. flavescens* group bacteria (Velmurugan et al. 2016) as also shown very recently (du Toit et al. 2024); however, effects on *Rothia* spp. were very minor there. The tongue community, which has not been analyzed in detail previously for its adaptation to nitrate consumption, offers a promising environment to analyze such effects as it harbors a clearly defined community not being modulated by bacteria shedding off from other sites such as the saliva. Our results on enrichment of *N. flavescens* group bacteria and *Abiotrophia* and depletion of different *Actinomyces* and *Stomatobaculum* are in agreement with changes observed recently in a Swedish clinical trial (du Toit et al. 2024)*.* However, neither the relative abundance of *Rothia* nor that of *Veillonella* were affected by juice consumption in our study. Importantly, all oral niches are inhabited by an enormous amount of facultative anaerobic bacteria capable of nitrate reduction and addition of a possible electron acceptor not necessarily means its use for energy production by bacteria capable to do so. On the other side, addition of a nitrate-containing nutrient may also add other bioactive compounds, which could induce nitrate-independent changes. In fact, a recent study comparing the effects of diets comprising nitrate rich green leafy vegetables versus potassium nitrate could show a significant effect due to nitrate as exemplified by the enrichment of *Neisseria* species and reductions in *Veillonella* (du Toit et al. 2024). Green leafy vegetables, however, were shown to have a more pronounced effect on altering the composition of the oral microbiome compared with potassium nitrate and the nitrate-independent increase in *Rothia* and decrease in *Actinomyces* could be related to the presence of other bioactive compounds (du Toit et al. 2024). Thus, complex microbial interactions and competitions for electron acceptors and donors as well as bioactive compounds present in nutrients may be a reason for on the first view inconsistent results reported.

To our knowledge, the influence of nitrate administration on subgingival communities has only seldomly been described. Lettuce juice consumption was reported to result in an increase in *Neisseria* and *Rothia* organisms (Jockel-Schneider et al. 2021) as indicated for saliva. The PERMANOVA analysis performed here, however, indicated the most severe effect of juice administration on subgingival rather than tongue or saliva communities, even though these differences were not mirrored in single genera or species being differentially distributed. This indicates changes in the complete microbial net of interacting species.

Some of the taxa indicated by us to be enriched or depleted after nitrate consumption in subgingival samples have already been reported to react in saliva such as *C. showae*, *C. leadbetteri*, and *C. sputigena.* However, taxa such as *Corynebacterium* (and the species *C. matruchotii*), the enrichment of which during orthodontic treatment was prevented by juice consumption, are specific for the subgingival niche (Mark Welch et al. 2016). Interestingly, *C. matruchotii* is documented to form a physical bridge between the base of the gingival biofilm and its outer layers (Mark Welch et al. 2016), and thus, prevention of its enrichment directly interferes with biofilm formation.

In conclusion, fixed orthodontic treatment influences subgingival, tongue and saliva microbiota, and clinical parameters over time. Our study revealed that the composition of all communities changed significantly during the 6 months after insertion of fixed orthodontic appliances. However, subgingival communities reacted promptly to orthodontic treatment with an enrichment of periodontal pathogens, whereas saliva and tongue showed a delayed response. Daily administration of nitrate-rich dietary supplements influenced specifically the subgingival microbiota also by counteracting effects exerted by orthodontic treatment. Overall, the effects of dietary supplements are associated with the sampling site in the oral cavity which should be considered in further clinical settings.

There are some limitations in the present study. The present pilot study includes a small sample size of patients which specifically hampers the analysis of the effect of juice application. Moreover, there is a certain bias in the control versus the juice consuming group concerning age (14.5 ± 1.6 versus 12.5 ± 1.1 y) and specifically gender (18% versus 72% of females), which is visible in some minor differences in the microbial communities at t0. These differences are, however, not reflected in the PERMANOVA analysis (Table 2). An effect of gender on oral microbial communities has in fact been observed previously (Minty et al. 2021; Ortiz et al. 2019) and has to be taken to consideration in future studies, which also should evaluate larger cohorts.

## Supplementary Information

Below is the link to the electronic supplementary material.

Supplementary file1 (XLSX 19769 KB)

Supplementary file2 (PDF 3274 KB)

## Data Availability

Demultiplexed raw data for all the amplicon sequencing pair-end datasets are publicly available at the NCBI Sequence Reads Archive (SRA) under BioProject accession number PRJNA1158742 (https://dataview.ncbi.nlm.nih.gov/object/PRJNA1158742?reviewer=6fu6oh2pu2i48f1obgidttpkvu).
