# Supplementary material for: Influence of orthodontic appliances and nitrate on the oral microbiota
Source: Appl Microbiol Biotechnol. 2025 May 6;109:111. doi: 10.1007/s00253-025-13496-0 (PMC12055954; doi:10.1007/s00253-025-13496-0)
Supplement: Supplementary file 1 [file 253_2025_13496_MOESM2_ESM.pdf]

Applied Microbiology and Biotechnology

Supplementary Material (Supplementary Figures and Tables)

Influence of orthodontic appliances and nitrate on the oral microbiota

Elisabeth Reichardt[1,4], Martin Eigenthaler[2], Paul-Georg Jost-Brinkmann[3], Angelika Stellzig-Eisenhauer[2], Carlalberta Verna[4], Iris Plumeier[5], Silke Kahl[5], Howard Junca[5], Ramiro Vilchez-Vargas[6], Dietmar H. Pieper[5]

[1]Department of Dentistry and Oral Health, Aarhus University, Aarhus, Denmark

[2]Department of Orthodontics, University Hospital Würzburg, Würzburg, Germany

[3]Department of Orthodontics, Dentofacial Orthopedics and Pedodontics, CharitéCenter for Oral Health Sciences CC3, Charité - Universitätsmedizin Berlin, Berlin, Germany

[4]Department of Pediatric Dentistry and Orthodontics, University Center of Dental Medicine, UZB, Basel, Switzerland

[5]Microbial Interactions and Processes Research Group Helmholtz Centre for Infection Research, Braunschweig, Germany

[6]Medical Department 2, University Hospital LMU Munich, Munich, Germany

Correspondence: Elisabeth Reichardt, Department of Dentistry and Oral Health, Aarhus University, Vennelyst Boulevard 9, 8000 Aarhus, Denmark, e-mail: elisabeth.reichardt@dent.au.dk

Table of Contents

Supplementary Table S1 Age and sex at t0 of the 22 healthy participants before undergoing orthodontic treatment

Supplementary Table S4 Factors influencing global community structures as indicated by PERMANOVA.

Supplementary Figure S1 Bacterial community diversity in saliva, SA; dorsum of the tongue, TO; and subgingival plaque, SU samples.

Supplementary Figure S2 Bacterial community richness in SA, TO and SU samples over time and depending on juice application.

Supplementary Figure S3 Bacterial community diversity in SA, TO and SU samples over time and depending on juice application.

Supplementary Figure S4 Bacterial community evenness in SA, TO and SU samples over time and depending on juice application.

Supplementary Table S1 Age and sex at t0 of the 22 healthy participants before undergoing orthodontic treatment. F = female; M = male.

Groups	Age (mean ± SD) at t0	Sex at t0
Control group	14.45 ± 1.57	2 F, 9 M
Juice group	12.45 ± 1.12	8 F, 3 M

Supplementary Table S4 Factors influencing global community structures as indicated by PERMANOVA. The significance of differences in community structure between different sampling sites, time and juice application was calculated by PERMANOVA (main test). The Pseudo-F and the *p*-values are given for each factor performed at different taxonomic levels (from sequence type to genus). The *t* statistics and the *p*-values are also given for paired tests among different sites and times. Analysis was performed at different taxonomic levels (from sequence type to genus). Significant *p*-values are shown in bold.

	Sequence type		Species		Genus	
Factor	Pseudo-F	*p*	Pseudo-F	*p*	Pseudo-F	*p*
Site	12.192	**0.001**	29.194	**0.001**	24.192	**0.001**
Time	1.580	**0.003**	3.124	**0.001**	3.995	**0.001**
Juice	2.822	**0.001**	2.698	**0.003**	2.927	**0.010**
Site x time	0.688	1	1.375	**0.036**	2.237	**0.001**
Site x juice	1.032	0.357	1.473	0.058	1.408	0.120
Time x juice	0.920	0.671	0.907	0.566	1.117	0.331
Groups compared	*t*	*p*	*t*	*p*	*t*	*p*
SA, SU	3.373	**0.001**	5.083	**0.001**	5.046	**0.001**
SA, TO	2.159	**0.001**	3.433	**0.001**	2.492	**0.001**
SU, TO	4.404	**0.001**	6.598	**0.001**	5.773	**0.001**
t0, t1	1.123	0.087	1.387	**0.024**	1.266	0.101
t0, t2	1.434	**0.003**	2.106	**0.001**	2.337	**0.001**
t1, t2	1.194	**0.039**	1.747	**0.001**	2.275	**0.001**

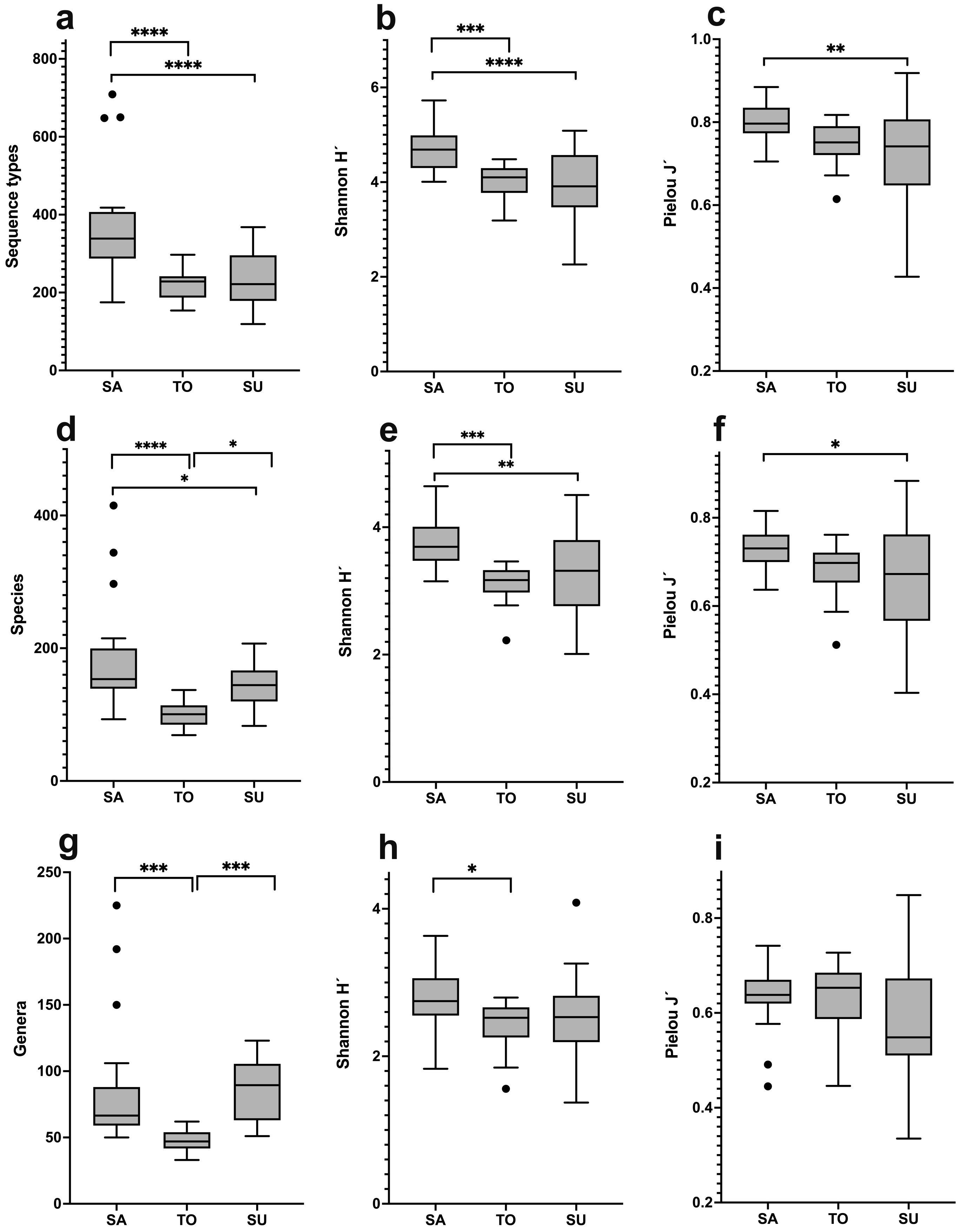

Supplementary Fig. S1 Bacterial community diversity in saliva, SA; dorsum of the tongue, TO; and subgingival plaque, SU samples. Diversity is indicated by total taxon numbers, Shannon diversity (H'), Pielou's evenness (J'), respectively, and was analyzed using sequence type (a-c), species (d-f) and genus (g-i) relative abundance data as input. Statistically significant differences are indicated as *$p<0.05$, **$p<0.01$, ***$p<0.001$ or ****$p<0.0001$. The mean is indicated by a black line. The box represents the interquartile range. The whiskers extend to the upper adjacent value (largest value = 75th percentile + 1.5 x IQR) and the lower adjacent value (lowest value = 25th percentile - 1.5 x IQR) and the dots represent outliers.

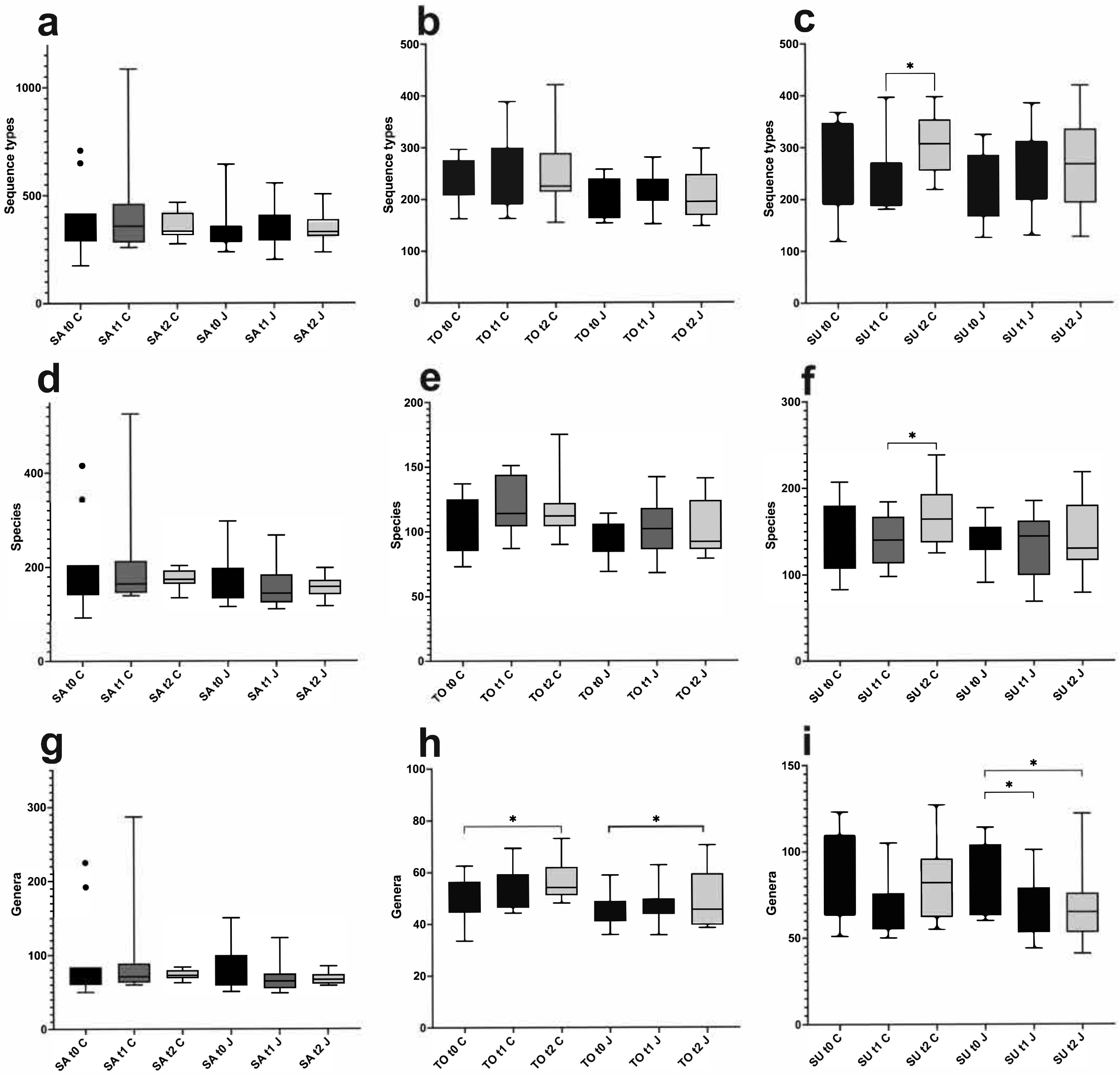

Supplementary Fig. S2 Bacterial community richness in SA, TO and SU samples over time and depending on juice application. Richness is indicated by total taxon numbers and was analyzed using sequence type (a-c), species (d-f) and genus (g-i) relative abundance data as input. Statistically significant differences are indicated as *$p<0.05$. The mean is indicated by a black line. The box represents the interquartile range. The whiskers extend to the upper adjacent value (largest value = 75th percentile + 1.5 x IQR) and the lower adjacent value (lowest value = 25th percentile - 1.5 x IQR) and the dots represent outliers.

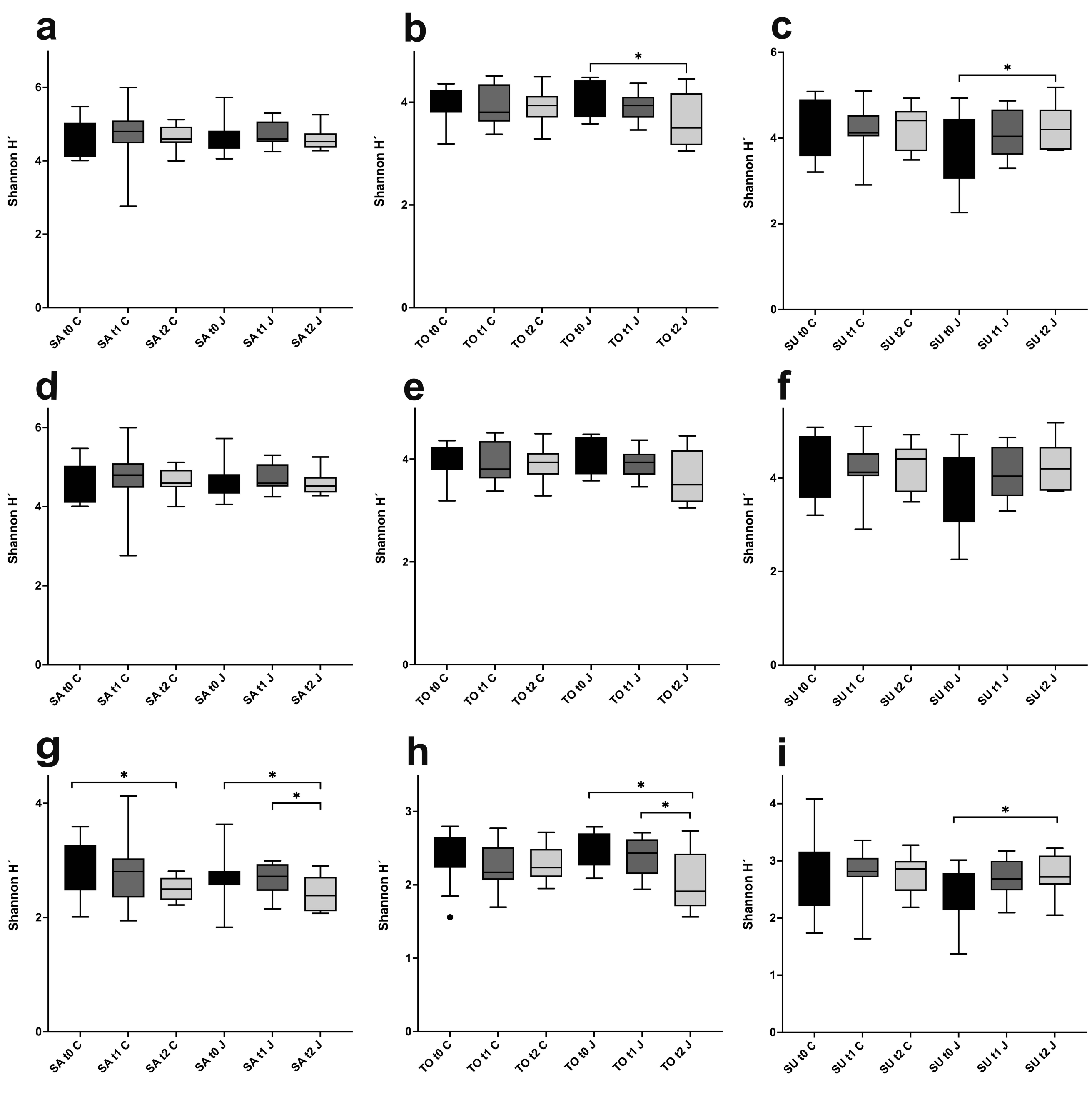

Supplementary Fig. S3 Bacterial community diversity in SA, TO and SU samples over time and depending on juice application. Diversity is indicated by Shannon diversity (H') and was analyzed using sequence type (a-c), species (d-f) and genus (g-i) relative abundance data as input. Statistically significant differences are indicated as **p*<0.05. The mean is indicated by a black line. The box represents the interquartile range. The whiskers extend to the upper adjacent value (largest value = 75th percentile + 1.5 x IQR) and the lower adjacent value (lowest value = 25th percentile - 1.5 x IQR) and the dots represent outliers.

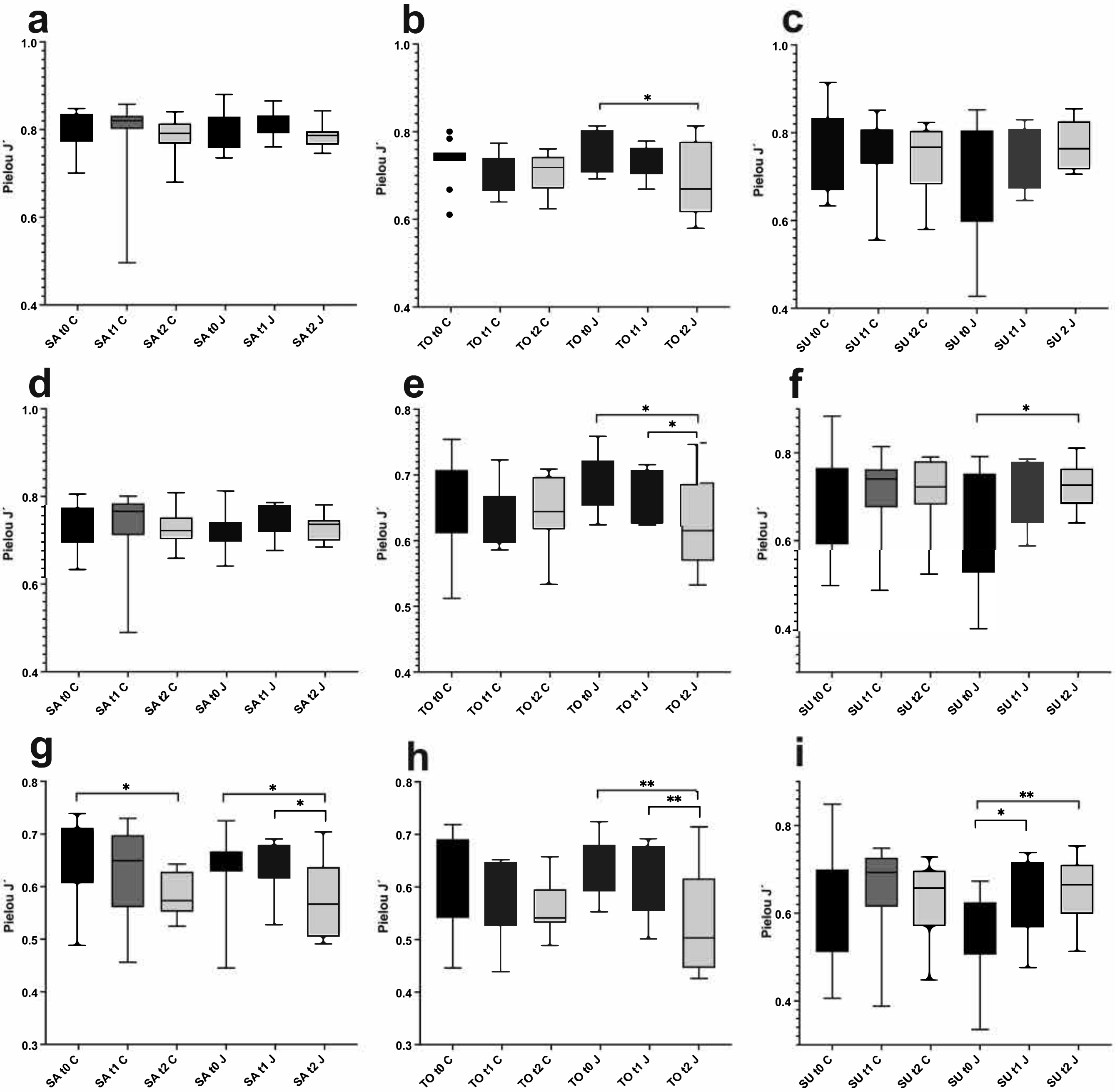

Supplementary Fig. S4 Bacterial community evenness in SA, TO and SU samples over time and depending on juice application. Evenness is indicated by Pielou's evenness (J') and was analyzed using sequence type (a-c), species (d-f) and genus (g-i) relative abundance data as input. Statistically significant differences are indicated as **p*<0.05. The mean is indicated by a black line. The box represents the interquartile range. The whiskers extend to the upper adjacent value (largest value = 75th percentile + 1.5 x IQR) and the lower adjacent value (lowest value = 25th percentile - 1.5 x IQR) and the dots represent outliers.
